# Adductor canal block versus femoral nerve block for pain control after total knee arthroplasty: A systematic review and Meta-analysis

**DOI:** 10.1097/MD.0000000000030110

**Published:** 2022-08-26

**Authors:** Elfatih A. Hasabo, Ahmed Assar, Maysa Madny Mahmoud, Hamid Ali Abdalrahman, EzzElDien A. Ibrahim, Menna Allah Hasanin, Amr Khaled Emam, Yossef Hassan AbdelQadir, Ahmed Alaa AbdelAzim, Ahmed Said Ali

**Affiliations:** a Faculty of Medicine, University of Khartoum, Khartoum, Sudan; b International Medical Research Association (IMedRA); c Faculty of Medicine, Menofia University, Shebin El kom, Menofia, Egypt; d Faculty of Medicine, South Valley University, Qena, Egypt; e Faculty of Medicine, University of Gezira, Wad Madani, Sudan; f Faculty of Medicine, Cairo University, Cairo, Egypt; g Faculty of Medicine, Al-Azhar University of Cairo, Cairo, Egypt; h Faculty of Medicine, Alexandria University, Alexandria, Egypt; i Faculty of Medicine, Beni Suef university, Egypt.

**Keywords:** adductor canal block, femoral nerve block, postoperative analgesia, total knee arthroplasty

## Abstract

Femoral nerve block is a widely accepted nerve block method with evident reduction in consumption of opioid painkiller and minimization of the duration of hospital stay but may cause weakness of quadriceps muscle strength. Adductor canal block is another nerve block technique that attracts the attention of scientific community nowadays because of its possible superiority over Femoral nerve block regarding mobility and muscle strength.

**Methods:**

This is a systematic review and meta-analysis of 33 studies, aiming to compare femoral nerve block with adductor canal block following total knee arthroplasty regarding pain control and mobilization.

**Results:**

Adductor canal block showed better preservation of quadriceps muscle strength (MD = 0.28, 95% CI [0.11, 0.46], *P* = .002), and better mobilization up to 2 days postoperatively. However, no significant difference was found between the 2 interventions regarding pain control (MD = 0.06, 95% CI [−0.06, 0.17], *P* = .33) or opioid consumption (SMD = 0.08, 95% CI [−0.06, 0.22], *P* = .28) up to 2 days postoperatively. The better mobilization results of adductor canal block did not translate into a significant difference in the risk of falls or patients’ satisfaction; however, adductor canal block patients had less mean length of hospital stay than the patients with femoral nerve block.

**Conclusion:**

Both femoral nerve block and adductor canal block provide similar results regarding pain control and opioid consumption, however adductor canal block provides better preservation of quadriceps strength and mobilization, giving it more advantage over femoral nerve block.

## 1. Introduction

Total knee arthroplasty (TKA) is a popular and effective surgical intervention for the treatment of knee osteoarthritis.^[1]^ The number of TKA operations has prominently increased over the last decade to be the most frequent surgical operation done in the developed world.^[2]^ However, TKA is known to cause moderate to severe postoperative pain that delays the recovery process.^[3]^ The pain following TKA increases the patients’ risk to various postoperative complications including infections, loosening of the joint, reflex sympathetic dystrophy and immobility-related complications as deep venous thrombosis (DVT).^[3,4]^

Peripheral nerve blocks (PNBs) are analgesic techniques used after TKA primarily for pain control. In addition to pain reduction, nerve blocks significantly enhance recovery and reduce both hospital length of stay (LOS) and risk of re-admission.^[5]^ Femoral nerve block (FNB) is a widely accepted nerve block technique after TKA with high success rates in reduction of opioid consumption and minimization of the length of hospital stay.^[3,6]^ However, (FNB) may cause reduction of the quadriceps muscle strength impairing postoperative ambulation which increases the patients’ risk of falls after the surgery.^[7,8]^ Adductor canal block (ACB) is another nerve block technique that attracts the attention of the scientific community nowadays because of its possible superiority over (FNB).^[9]^ Several studies have documented that (ACB) is better than (FNB) regarding postoperative quadriceps muscle strength preservation, postoperative ambulation and functional recovery without any alteration of pain control.^[10–13]^ But on the contrary, 2 recent studies concluded that there is no statistically significant difference between ACB and FNB regarding the analgesic effect, quadriceps strength or functional recovery postoperatively.^[14,15]^

This systematic review aims to investigate the clinical efficacy of (ACB) compared to (FNB) and draw conclusions on whether or not ACB is superior to FNB regarding functional recovery without alteration of postoperative pain control following TKA.

## 2. Methods and Materials

We conducted this systematic review according to the Cochrane handbook for systematic reviews of interventions.^[16]^ Also, we reported this study using the preferred reporting items for systematic review and meta-analysis (PRISMA statement).^[17]^ This current review tests the hypothesis that patients with ACB will have better postoperative functional recovery, and muscle strength with–at least- same level of pain control as FNB. Ethical approval was not necessary for this study; because all data were obtained from previous published studies.

### 2.1. Search strategy

We searched PubMed, SCOPUS, web of science and Cochrane databases by using the keywords (Adductor canal block OR motor sparing knee blocks) AND (Femoral nerve block) AND (total knee arthroplasty OR total knee replacement) from conception till March 2021.

### 2.2. Eligibility criteria and study selection

We included only original papers (Randomized controlled trials or Cohort studies) which compare Adductor canal block with femoral nerve block in total knee arthroplasty patients and excluded any review, case report, systemic review, meta-analysis, or animal studies as well as studies with data that cannot be extracted. Reviewers independently screened the retrieved citations in 2 steps; title and abstract screening followed by full text screening.

### 2.3. Data extraction

Authors extracted the following data from the included studies:

1. Baseline characters of the studies’ participants and summary of the included studies,
2. Study outcomes: pain control measured by visual analog scale (VAS) at rest and at motion—Quadriceps muscle strength (knee extensors strength) by Isometric measurement or manual muscle testing (MMT)–Mobilization after the operation measured by timed up and Go test and ambulation distance—the amount of Opioid consumption - length of hospital stay—Risk of falls - patient satisfaction.

### 2.4. Quality Assessment

We assessed the Quality of included trials using Cochrane Risk of Bias tool provided in Cochrane handbook for systematic reviews of interventions (version 5.1.0).^[16]^ The domains included were: (1) Random sequence generation (selection bias). (2) Allocation concealment (selection bias). (3) Blinding of participants and personnel (performance bias). (4) Outcomes assessment (detection bias). (5) Incomplete outcome data (attrition bias). (6) Other potential sources of bias. The reviewers judged the domains as: “ low risk,” “high risk,” or “ unclear”. The quality assessment table used was provided in (part 2, chapter 2.5) of the same book.^[16]^ The quality of the included cohort studies was assessed by the quality assessment tool of the National Heart, Lung, and Blood Institute (NHLBI).^[18]^ We used the tool for observational cohort studies and cross-sectional studies. This tool is composed of 14 questions to assess the risk of bias and confounders. These questions were answered by “yes,” “no,” “cannot determine, ” “not applicable,” or “not reported” then each study was given a score to guide the overall rating of the quality as “good,” “fair,” or “poor” quality.

### 2.5. Data analysis

In the analysis, we presented the dichotomous data as risk ratio (RR) and continuous data as mean difference (MD) or standard mean difference (SMD), in a random-effects meta-analysis model using the inverse-variance method for continuous data and Mantel-Haenzel method for dichotomous data. Missing SD was calculated from standard error or 95% confidence interval (CI) according to Altman.^[19]^ In this analysis, we used review manager 5.3 for windows.

### 2.6. Assessment of heterogeneity

The heterogeneity of the pooled data was assessed by I square and chi-square tests presented in the forest plots. The chi-square test measures the presence of significant heterogeneity. And the I-square test quantifies the size of the heterogeneity in the pooled data. Interpretation of the results followed the recommendations of the Cochrane handbook for systematic reviews and meta-analysis. The chi-square test was considered significant with a *P* value less than (.1) and the I-square test was interpreted as follows: ((0–40 %): might not be important; (30–60%): may represent moderate heterogeneity; (50–90 %): may represent substantial heterogeneity).

## 3. Results

### 3.1. Literature search

**Figure 1.**
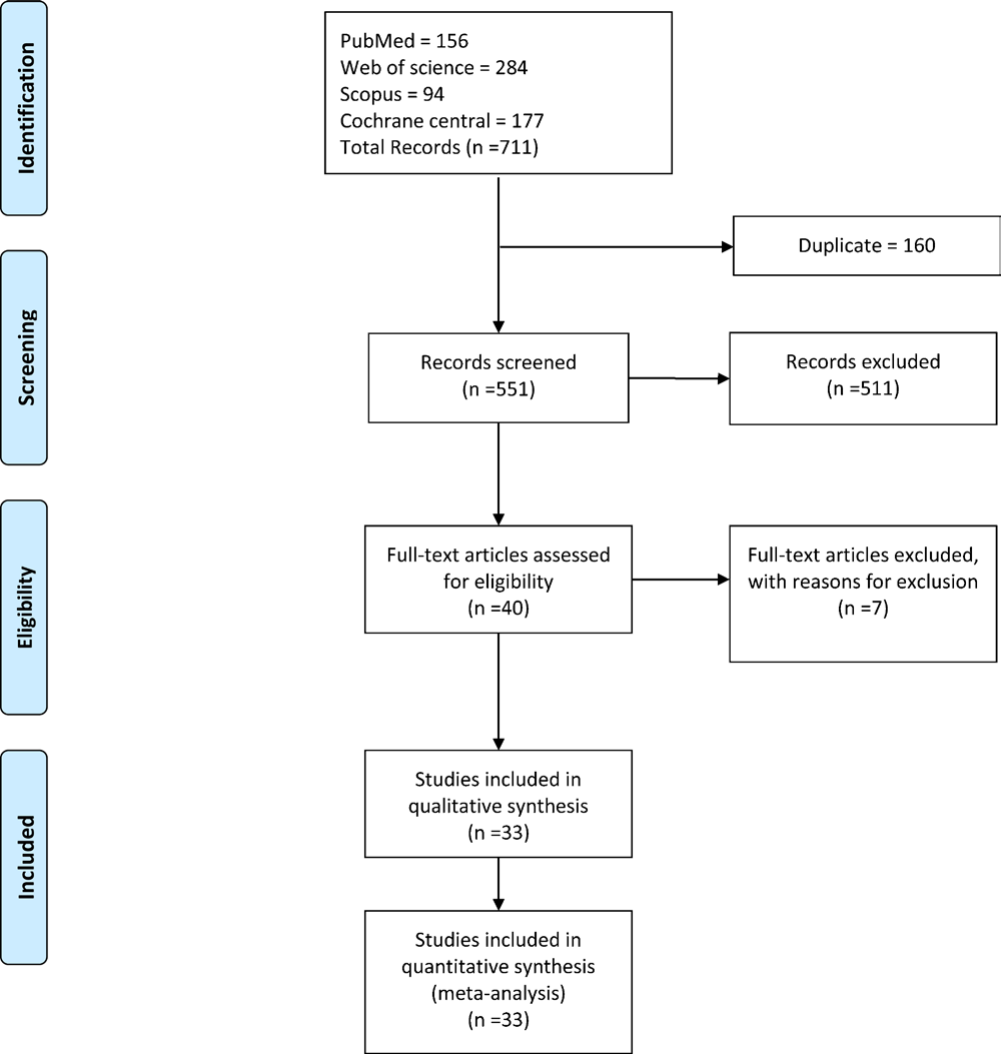
PRISMA flow chart.

The literature search retrieved 711 citations. After title and abstract screening, 40 articles were selected. We evaluated the full text of the selected studies. Finally, 33 studies were eligible to be included in our review and quantitative analysis (PRISMA flow diagram; Fig. 1).

### 3.2. Characteristics of the included studies and quality assessment

**Figure 2.**
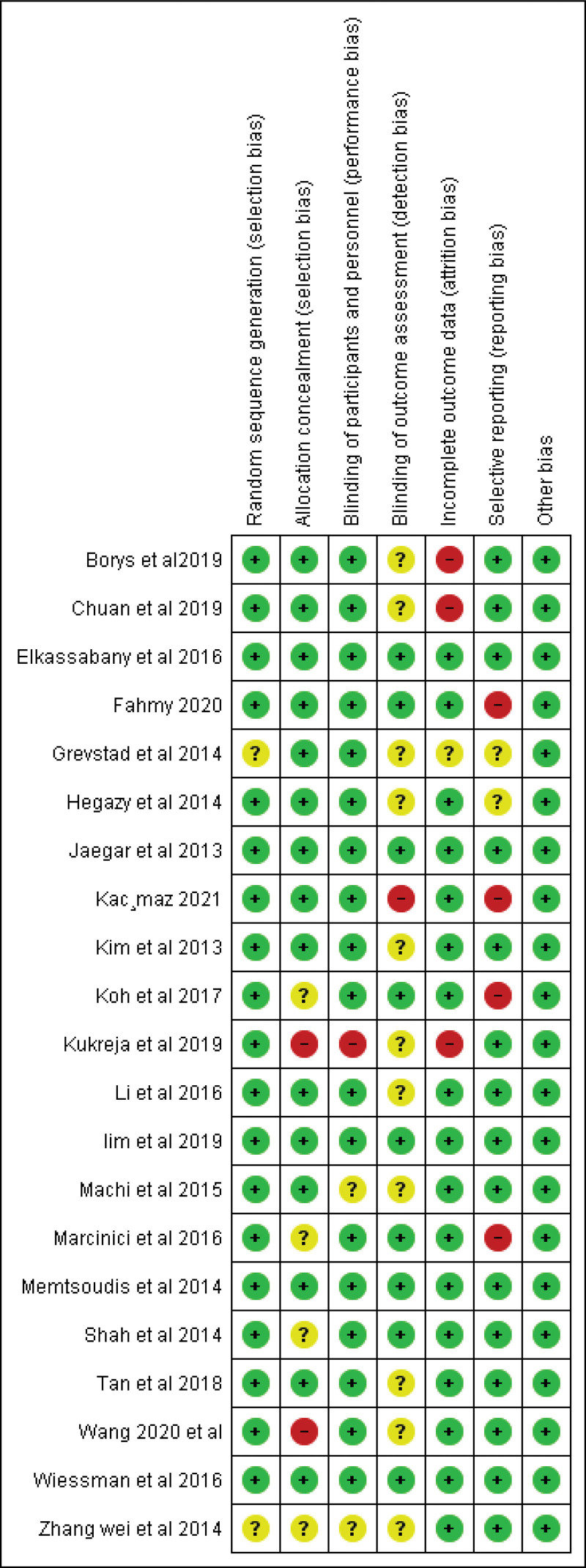
Risk of bias summary.

The baseline characteristics of the studies’ participants are shown in (Table 1) and the summary of all the included studies is present in (Supplementary table 1, http://links.lww.com/MD/G1000). A summary of the quality assessment for the included randomized trials is shown in (Fig. 2), All the included randomized controlled trials showed moderate to high quality. Eleven cohort studies^[24,25,27,28,34–38,40,41]^ were fair in quality according to NIH (national institute of health) quality assessment tool for Observational Cohort. One study^[42]^ had good quality. For more details and answers to all assessment questions in each study, see Supplementary table 2, http://links.lww.com/MD/H2.

**Table 1:** Baseline characters of the studies’ participants.

Study ID	Groups	No of patients	Age (yr)	Male (%)	Body mass index	Duration of surgery (min)
Fahmy et al 2020.^[20]^	ACB	40	59.5 ± 4.6	14	24.4 ± 4.6	100.1 ± 2.9
	FNB	40	60.1 ± 1.1	13	23.5 ± 2.9	100.4 ± 3.1
Kac¸maz et al 2021.^[21]^	ACB	43	64.4 ± 1.7	20	–	–
	FNB	43	66.0 ± 1.4	25	–	–
Jaegar et al 2013^[22]^	ACB	23	70 ± 8	21.7	–	82 ± 20
	FNB	27	66 ± 9	51.8	–	75 ± 15
Kim et al 2013^[23]^	ACB	46	68 ± 9.4	47.8	29.9 ± 6.4	–
	FNB	47	67.6 ± 11.3	38.3	30.3 ± 5.8	–
Elkassabany et al 2016^[11]^	ACB	31	63 ± 8	29	31 ± 5	–
	FNB	31	65 ± 8	38.7	32 ± 6	–
Tan et al 2018^[13]^	ACB	100	64.2 ± 7.5	44	26.12 ± 3.6	71.5 ± 8.1
	FNB	100	63.5 ± 6.7	42	25.67 ± 2.88	72.6 ± 8.1
Ludwigson et al 2015^[24]^	ACB	148	64.09 ± 112	45.95	31.29 ± 75	–
	FNB	149	64.74 ± 112	44..97	31.48 ± 71	–
Seo et al 2017^[25]^	ACB	19	72.2 ± 5.3	21	–	–
	FNB	24	74.3 ± 6.81	16.67	–	–
Weissman et al 2016^[26]^	ACB	21	86.67 ± 11	42.85	29 ± 5.9	60 ± 18.5
	FNB	21	67.3 ± 8.89	43.85	31.3 ± 5.9	56.67 ± 11.1
Klement et al 2018^[27]^	ACB	118	65.5 ± 9.3	43.2	–	–
	FNB	146	66.8 ± 9.4	43.8	–	–
Mudumbai et al 2013^[28]^	ACB	66	65 ± 9	92.4	33 ± 6	105 ± 18
	FNB	102	66 ± 10	96	33 ± 7	105 ± 27
Machi et al 2015^[29]^	ACB	39	67 ± 8	41	30 ± 5	113 ± 32
	FNB	41	66 ± 7	34	29 ± 5	115 ± 21
Koh et al 2017^[30]^	ACB	50	64.3 ± 17.7	–	27.2 ± 10.14	–
	FNB	50				–
Grevstad et al 2014^[31]^	ACB	25	65.33 ± 28.88	28	–	71.6 ± 23.7
	FNB	25	63.33 ± 31.11	32	–	81 ± 45.92
Shah et al 2014^[32]^	ACB	48	68.31 ± 7.56	27.1	29.54 ± 5.46	68.85 ± 4.57
	FNB	50	65.94 ± 7.22	28	30.52 ± 5.3	68.30 ± 4.42
Marcinici et al 2016^[12]^	ACB	49	67 ± 8	39	31.5 ± 6	–
	FNB	49	67 ± 8	37	31.7 ± 5.4	–
Lim et al 2019^[15]^	ACB	15	63 ± 7	33	26.6 ± 4.3	–
	FNB	15	65 ± 8	47	28.0 ± 3.5	–
Hegazy et al 2014^[33]^	ACB	53	62 ± 12	47.2	31.3 ± 2.7	–
	FNB	54	63 ± 11	48.1	31.1 ± 2.8	–
Patterson et al 2015^[34]^	ACB	35	65.7 ± 8.9	31	35.3 ± 5.92	–
	FNB	41	65 ± 13.3	27	34.3 ± 9.6	–
Mudumbai et al 2015^[35]^	ACB	48	66.7 ± 13.3	100	33 ± 11.1	–
	FNB	46	67.3 ± 20	91	34.3 ± 12.6	–
Thacher et al 2017^[36]^	ACB	150	68.4 ± 31.1	21	33.8 ± 24.6	–
	FNB	129	68.8 ± 28.9	21	35.1 ± 28.4	–
Rassmussen et al 2014^[37]^	ACB	23	63.3 ± 11.9	20	34 ± 16.3	–
	FNB	22	62 ± 11.9	20	32.7 ± 10.4	–
Thobhani et al 2017^[38]^	ACB	22	64.3 ± 7.4	36.4	35.7 ± 9.6	–
	FNB	23	68.7 ± 6.7	39.1	33.3 ± 7.4	–
Memtsoudis et al 2014^[39]^	ACB	30	–	–	–	–
	FNB	29	–	–	–	–
Brennan et al 2018^[40]^	ACB	141	73.21 + 0.55	–	30.58 + 0.46	–
	FNB	104	72.28 + 0.78	–	31.47 + 0.57	–
Bolarinwa et al 2018^[41]^	ACB	791	–	–	–	–
	FNB	834	–	–	–	–
Ardon et al 2015^[42]^	ACB	45	64.86	31.1	–	93.31
	FNB	45	67.71	31.1	–	90.29
Li et al 2016^[43]^	ACB	24	62.3 ± 6.5	46	–	77.6 ± 8.2
	FNB	27	61.4 ± 6.8	48	–	76.6 ± 8.4
Zhang wei et al 2014^[44]^	ACB	30	63.7 ± 5.8	25	–	98.4 ± 10.3
	FNB	30	61.9 ± 6.7	36	–	97.1 ± 8.2
Kukreja et al 2019^[45]^	ACB	45	63.4	46.5	31.4	–
	FNB	45	65.4	46.3	32.3	–
Borys et al 2019^[10]^	ACB	43	67.33 ± 2.59	18.6	31.56 ± 1.85	–
	FNB	42	68.8 ± 2.37	19	30.8 ± 1.92	–
Chuan et al 2019^[14]^	ACB	75	66.66 ± 10.37	53	32.3 ± 4.37	91 ± 18.51
	FNB	76	68 ± 7.4	49	33.46 ± 7.03	96 ± 31.11
Wang et al 2020^[46]^	FTB	31	61.77 ± 3.66	50	–	87.77 ± 6.55
	ACB	32	61.67 ± 4.49	53	–	84.20 ± 6.10

ACB = adductor canal block, FNB = femoral nerve block, FTB = femoral triangle block.

### 3.3. Main outcomes of the study

**Figure 3.**
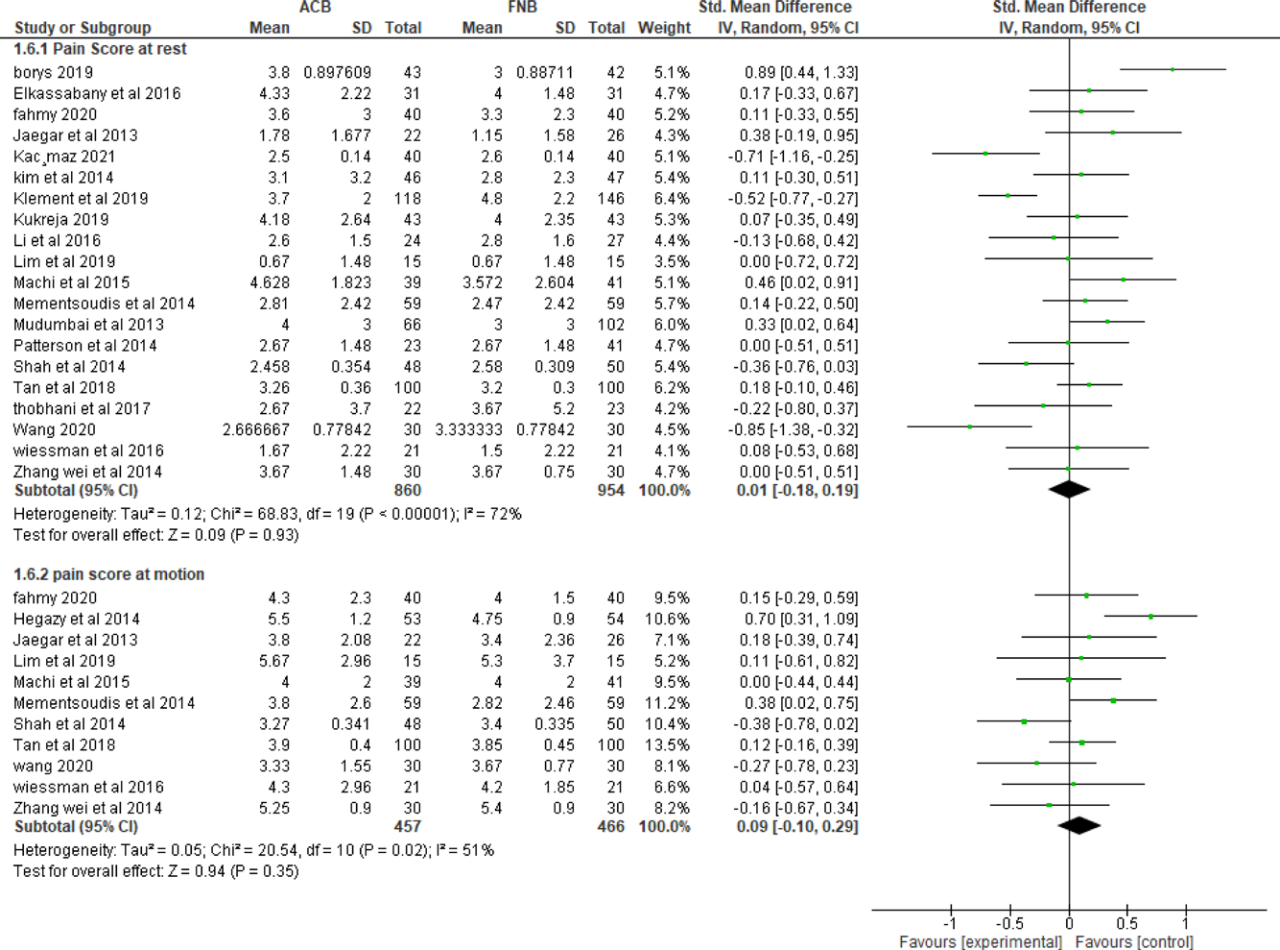
Pain Score at 24 hours.

**Figure 4.**
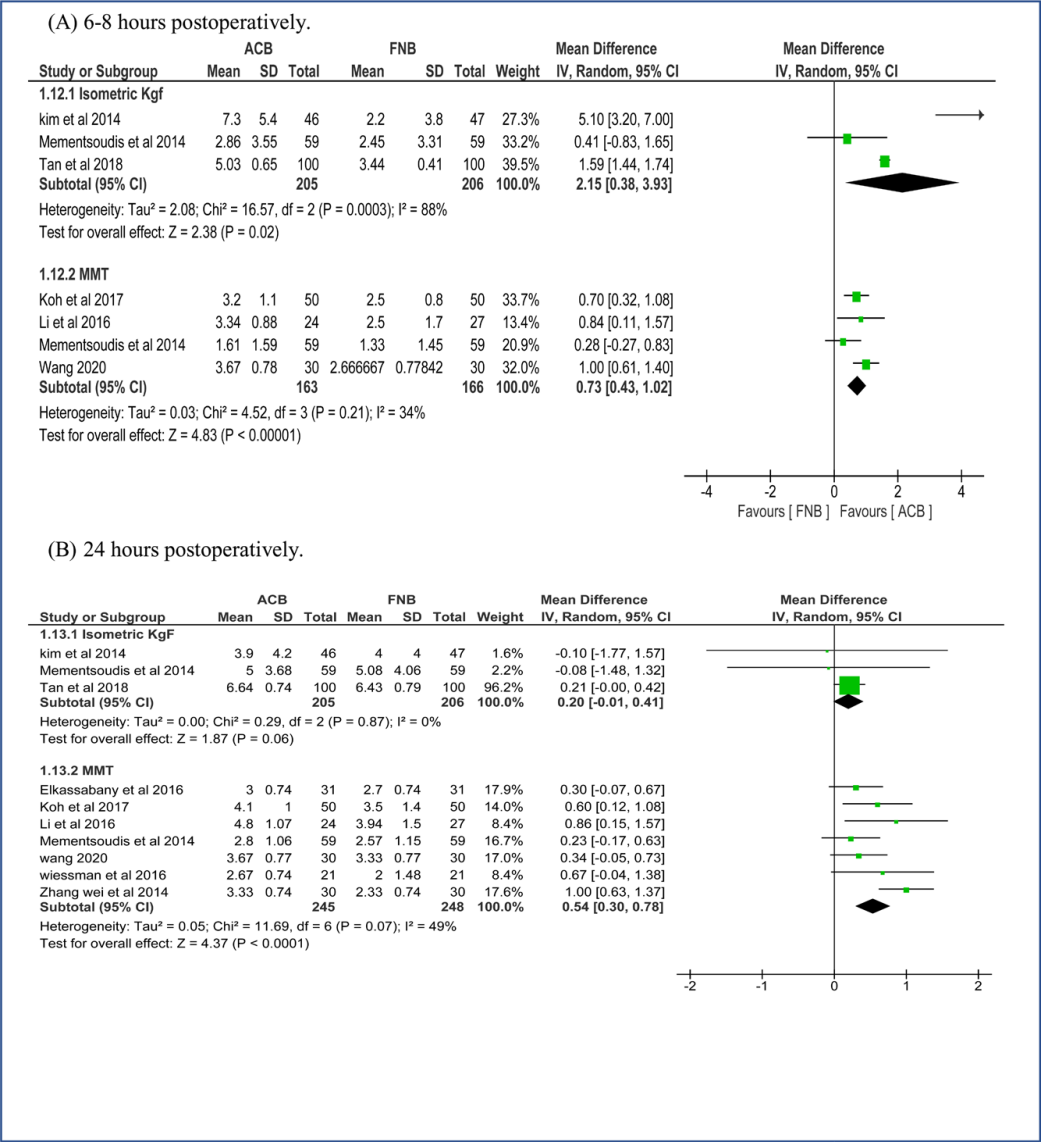
Quadriceps muscle strength: (A) at 6–8 hours postoperatively. (B) at 1 day postoperatively.

**Figure 5.**
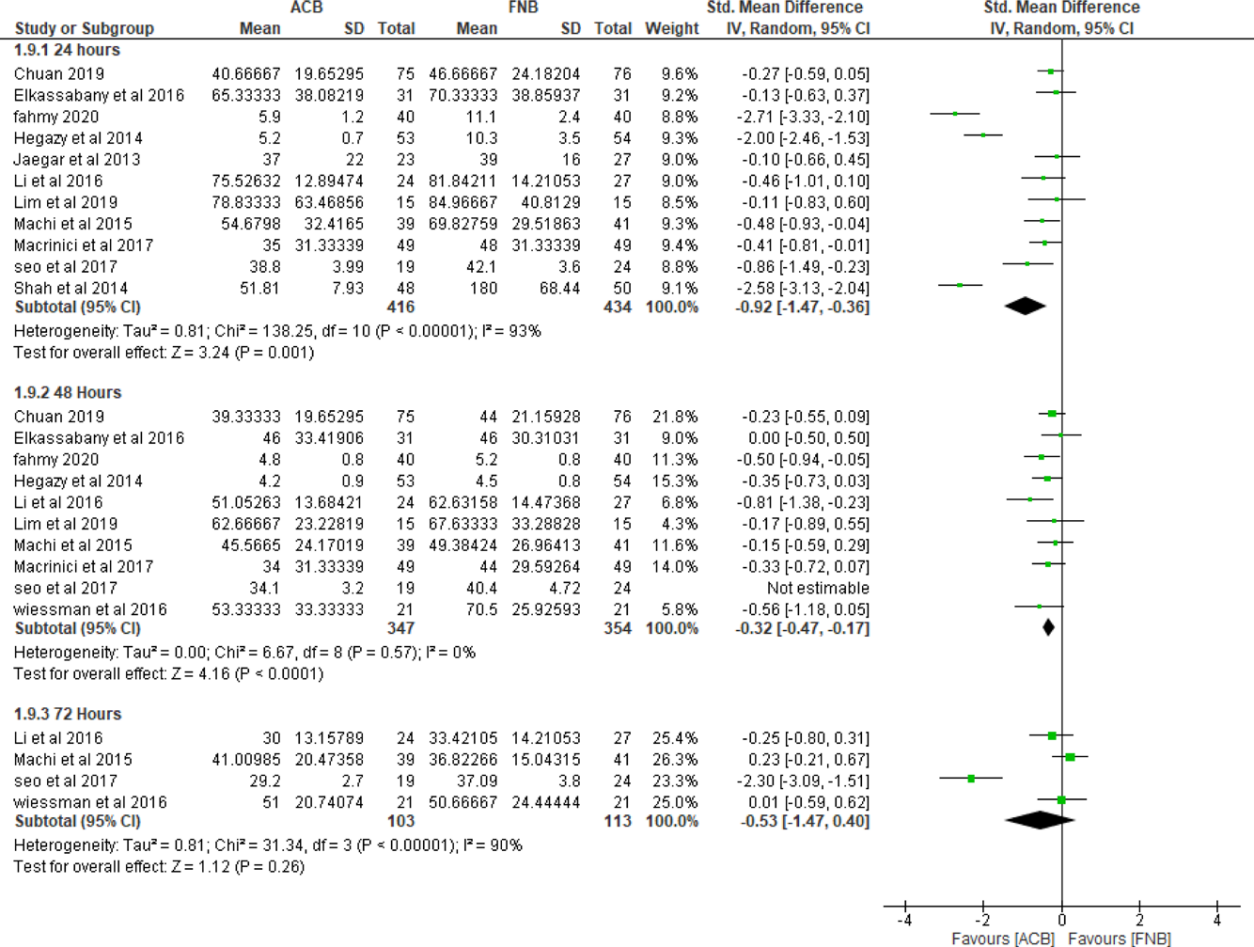
Mobilization by timed up and GO test TUG.

**Figure 6.**
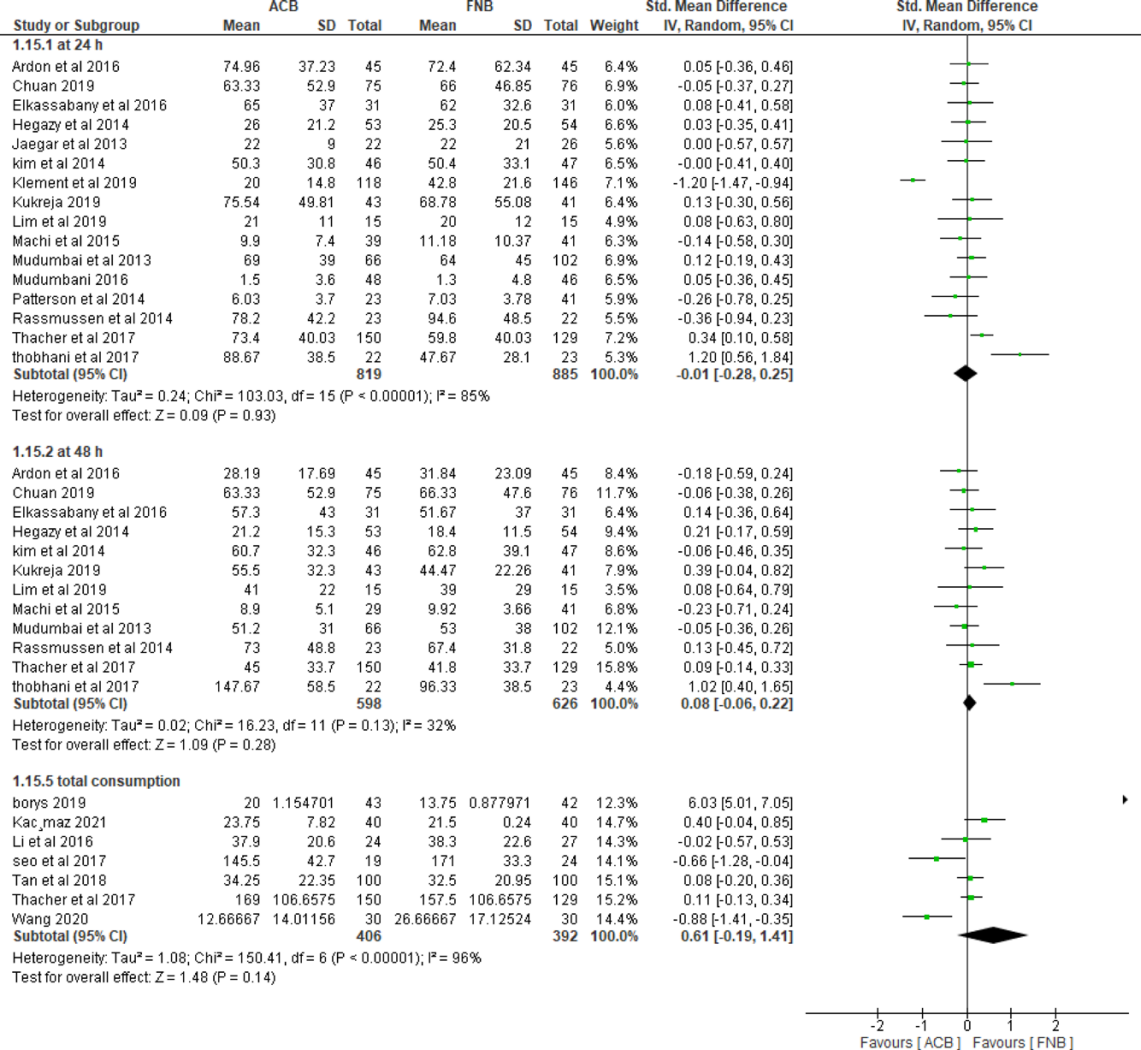
Opioid consumption.

A. Pain control measured by pain scores (VAS):
i. Pain scores at **6-8 hours at rest**
The pooled effect estimate showed no statistically significant difference between the 2 techniques at 6-8 hours (MD = -0.06, 95% CI [-0.45, 0.33], ***P*** = .77). (Supplementary figure 1, http://links.lww.com/MD/H3) Pooled results were heterogeneous (*P* < .00001, *I²* = 87%) and the detected heterogeneity could not be solved.
ii. Pain scores at **6-8 hours at motion**
The overall effect showed no statistically significant difference between the 2 interventions at motion (MD = -0.08, 95% CI [-0.47, 0.31], ***P*** = .70). (Supplementary figure 1, http://links.lww.com/MD/H3) Pooled results were heterogeneous (*P* < .0002, *I²* = 80%) and the detected heterogeneity was best resolved after excluding Wang et al (*P* = .78, *I²* = 0%) and the effect estimate remained nonsignificant (MD = 0.14, 95% CI [-0.03, 0.32], ***P*** < .11).
iii. Pain scores at **24 hours at rest**.
The pooled effect estimate showed no statistically significant difference between the 2 techniques at 24 hours postoperatively (MD = 0.01, 95% CI [-0.18, 0.19], *P* = .93). (Fig. 3). The pooled results were heterogeneous (*P* < .00001, *I²* = 72%), and heterogeneity could not be solved.
iv. Pain score at **24 hours at motion**.
The pooled studies showed no significant difference between the 2 interventions at motion (MD = 0.09, 95% CI [−0.10, 0.29], ***P*** = .35). The pooled results were heterogeneous (*P* = .02, *I²* = 51%) (Fig. 3). The detected heterogeneity could be solved by excluding Hegazy et al (*P* = .30, *I²* = 15%) and the effect estimate remained nonsignificant (MD = 0.03, 95% CI [−0.12, 0.18], *P* = .69).
v. Pain score at **48 hours at rest**.
The pooled effect estimate showed no statistically significant difference between adductor canal and femoral nerve block (MD = 0.06, 95% CI [−0.06, 0.17], *P* = .33). The studies were homogenous (*P* = .90, *I²* = 0%) (Supplementary figure 2, http://links.lww.com/MD/H4).
vi. Pain score at **48 hours at motion**.
The pooled studies showed no statistically significant difference between adductor canal and femoral nerve block (MD = 0.00, 95% CI [-0.13, 0.13], *P* = .99) (Supplementary figure 2, http://links.lww.com/MD/H4). The studies were heterogenous (*P* = .02, *I²* = 54%) and the detected heterogeneity was best solved by excluding Wang et al (*P* = .26, *I²* = 20%). Results remained nonsignificant (*P* = .42).
B. Quadriceps muscle strength:
i. Quadriceps muscle strength **6–8 hours postoperatively.**
**Isometric measurement**: ACB showed higher values of muscle strength over FNB on pooling means from included studies (MD = 2.15, 95% CI [0.38, 3.93], *P* = .02) (Fig. 4A). The pooled studies were heterogeneous (*P* = .0003, *I²* = 88%).
**MMT**: Pooled results showed that the ACB group has higher values of muscle strength (MD = 0.73, 95% CI [0.43, 1.02], *P* < .00001) (Fig. 4A). The pooled studies were homogenous (*P* = .21, *I²* = 34%).
ii. Quadriceps muscle strength **1 day postoperatively**.
**Isometric measurement**: pooled studies showed no statistically significant difference between the 2 interventions (MD = 0.20, 95% CI [-0.01, 0.41], *P* = .06) (Fig. 4B). The pooled studies were homogeneous (*P* = .87, *I²* = 0%).
**MMT**: Pooled results showed that the ACB group has higher mean values of muscle strength (MD = 0.54, 95% CI [0.30, 0.78], *P* < .0001) (Fig. 4B). The pooled studies were heterogonous (*P* = .07, *I²* = 49%).
iii. Quadriceps muscle strength **2 days postoperatively**.
**Isometric measurement**: pooled studies showed no statistically significant difference between the 2 interventions (MD = 0.05, 95% CI [-0.18, 0.28], *P* = .66) (Supplementary figure 3, http://links.lww.com/MD/H5). The pooled studies were homogeneous (*P* = .39, *I²* = 0%).
**MMT**: Pooled results showed that the ACB group has significantly higher mean values of muscle strength (MD = 0.28, 95% CI [0.11, 0.46], *P* = .002) (Supplementary figure 3, http://links.lww.com/MD/H5). The pooled studies were homogenous (*P* = .12, *I²* = 45%).
C. Mobilization after the operation
i. Mobilization by **ambulation and walking distance at 24 hours**.
The pooled mean difference showed that ACB significantly increases walking distance at 24 hours compared to femoral nerve block (MD = 46.32, 95% CI [13.77, 78.87], *P* = .005) (Supplementary figure 4, http://links.lww.com/MD/H6). The pooled studies were heterogeneous (*P* < .00001, *I²* = 99%) and the detected heterogeneity could not be solved by excluding a study.
ii. Mobilization by **ambulation and walking distance at 48 hours**.
The pooled mean difference showed that ACB significantly increases the walking distance at 48 hours compared to FNB (MD = 17.97, 95% CI [3.08, 32.86], *P* = .02). The pooled studies were heterogeneous (*P* = .002, *I²* = 68%) (Supplementary figure 4, http://links.lww.com/MD/H6).
iii. Mobilization by **timed up and GO test (TUG) at 24 hours**.
The pooled mean difference showed that ACB technique significantly decreases the test duration at 24 hours compared to FNB (SMD = −0.92, 95% CI [−1.47, −0.36], *P* = .001) (Fig. 5). The pooled studies were heterogeneous (*P* < .00001, *I²* = 93%). The detected heterogeneity could not be solved by excluding single study.
iv. Mobilization by **timed up and GO test (TUG) at 48 hours**.
The pooled mean difference showed that ACB technique significantly decreases the test duration at 48 hours compared to FNB (SMD = −0.41, 95% CI [−0.62, −0.20], *P* = .003) (Fig. 5). The pooled studies were heterogeneous (*P* = .04, *I²* = 49%). The detected heterogeneity could be solved by excluding Seo et al (*P* = .57, *I²* = 0%) and the effect estimate would remain significant (*P* = .0002).
v. **Mobilization** by **timed up and GO test (TUG) at 72 hours**.
The pooled mean difference showed no statistically significant difference between the 2 interventions at 72 hours (SMD = -0.53, 95% CI [-1.47, 0.40], *P* = .26) (Fig. 5). The pooled studies were heterogeneous (*P* < .00001, *I****²*** = 90%). The detected heterogeneity could be solved by excluding Seo et al (*I²* = 0%) and the effect estimate would remain nonsignificant (*P* = .80)
D. Opioid consumption
i. At **24 Hours**.
The pooled effect estimate showed no statistically significant difference between the 2 intervention groups (SMD = -0.01, 95% CI [-0.28, 0.25], *P* = .93) (Fig. 6). Pooled results were heterogeneous (*P* < .00001, *I²* = 85%) and the detected heterogeneity could be best solved by excluding klement et al 2019 (*P* = .08, *I²* = 37%). The pooled results would remain nonsignificant.
ii. At **48 Hours**.
The pooled effect estimate showed no statistically significant difference between the 2 intervention groups (SMD = 0.08, 95% CI [-0.06, 0.22], *P* = .28) (Fig. 6). Pooled results were homogenous (*P* = .13, *I²* = 32%).
iii. Total opioid consumption.
Results showed no statistically significant difference between the 2 intervention groups (SMD = 0.61, 95% CI [-0.19, 1.41], *P* = .14) (Fig. 6). Pooled results were heterogenous (*P* < .00001, *I²* = 96%) and it could not be solved.
E. Recovery After the operation:
i. Length of hospital stay.
Pooled results showed that ACB was associated with significantly lower period of hospital stay when compared to FNB (MD = −0.25, 95% CI [-0.48, −0.02], *P* = .04) (Supplementary figure 5, http://links.lww.com/MD/H7). Pooled results were heterogeneous (*P* < .00001, *I²* = 92%) and the detected heterogeneity could not be solved by excluding any study.
ii. Risk of falls
The pooled results showed no statistically significant difference between ACB and FNB regarding the risk of postoperative falls (MD = 1.09, 95% CI [0.77, 1.53], *P* = .64) (Supplementary figure 6, http://links.lww.com/MD/H8). Pooled results were heterogeneous (*P* = .03, *I²* = 67%) and the detected heterogeneity could be solved by excluding Bolarina et al (*I²* = 18%). And the effect estimate would remain nonsignificant.
iii. Mean patient satisfaction
The pooled results showed no statistically significant difference between ACB and FNB (MD = 0.08, 95% CI [-0.06, 0.22], ***P*** = .28) regarding the patients’ satisfaction (Supplementary figure 7, http://links.lww.com/MD/H9). Pooled results were homogeneous (*P* = .21, *I²* = 27%).

## 4. Discussion

The pooled results of the studies included in this meta-analysis showed that both ACB and FNB exhibit equal pain control and opioid consumption after 24 and 48 hours of total knee arthroplasty operation both in rest and in motion; however, ACB showed superiority to FNB regarding quadriceps muscle strength up to 2 days postoperation specially when assessing the muscle strength using manual muscle testing. ACB also showed better mobilization results than FNB up to 2 days postoperation but equal results after 3 days, the better mobilization results did not translate into any difference in the risk of falls or patients’ satisfaction about the procedure.

Total Knee arthroplasty is a successful surgical procedure with excellent long-term survival rates.^[47–49]^ The main aim of patients undergoing TKA is to alleviate pain and improve their functional mobility, thus no leniency is allowed in handling such aspect of the patient complaint.^[50]^ Besides suffering and discomfort, severe unrelieved postoperative pain delays rehabilitation and lengthens the hospital-stay period, and may lead to persistent postsurgical pain.^[51]^ Previous studies reported poor management and a higher percentage of patients with severe pain after TKA procedure.^[52,53]^ Almost 44–57% of the patients who have undergone the surgery are woken up by pain during the first 3 days after TKA.^[53]^ The developed sleep deprivation reduces pain threshold generating a vicious cycle and causing dissatisfaction in about 19% of the patients undergoing TKA.^[54,55]^ Therefore, sleep disturbance and persistent postoperative pain appear to be crucial predictors of persistent functional limitations at 1 and 3 months after TKA.^[54,56]^

Our nonsuperiority results between both modalities of anesthesia in TKA in terms of pain control and opioid consumption are in line with the results of many other studies.^[14,15,57,58]^ A recent meta-analysis showed an equivalent effect of ACB and FNB in patients with TKA.^[59]^ Lim et al found that the perioperative morphine consumption and pain scores at 1, 24, and 48 hours postoperatively were similar between the groups.^[15]^ Likewise, both Kim et al and Jaeger et al showed that pain scores and opioid consumption were similar in both ACB and FNB groups.^[22,23]^ Moreover, the superiority of ACB over FNB in terms of mobility and muscle strength is also consistent with the results reported in previous trials.^[11,31,60]^ ACB is proposed to have a quadriceps-sparing effect, as it blocks distally to where most of the motor fibers of the femoral nerve branch off.^[61]^ Both Jaeger et al and Kwofie et al showed preservation of quadriceps strength with ACB as opposed to FNB.^[8,62]^ Jaeger et al reported quadriceps strength of 52% of the baseline value in patients with continuous ACB and 18% only in patients with continuous FNB.^[22]^

Regarding the risk of falls, we found no statistical difference between both interventions in the meta-analysis model. nevertheless, Kwofie et al (using the Berg Balance Scale) demonstrated a higher incidence of quadriceps muscle weakness and risk of falls after administration of FNB.^[62]^ Elkassabany et al used the Tinetti Scale for gait and balance to report a higher incidence of falls in the FNB group after 48 hours.^[11]^ The weakness of quadriceps with FNB was also demonstrated by Thacher et al who reported a statistically significant difference in episodes of near fall (knee-buckling) in about 13% of patients with FNB vs 2% with ACB during physiotherapy.^[36]^

Despite all the aforementioned advantages of the 2 peripheral nerve blockade techniques being investigated in this study, various limitations and disadvantages may exist. Patients undergoing peripheral nerve blockades carry the risk for a possible nerve injury during the procedure, in addition to possible local and systemic toxicities from the large volume of local anesthetic used in the procedure.^[63,64]^ Despite its advantage in preservation of muscle strength postoperatively, ACB carries an increased risk for neuropathy, myositis, and infection due to perioperative injection of local anesthetic in the adductor canal close to the operative site, in addition to ischemia resulting from possible tourniquet compression.^[65]^ Another major challenge in the use of ultra-sound guided peripheral nerve blockade techniques, is the requirement of a highly skilled physician to perform the procedure specifically in cases of smaller and deeper nerves, or in individuals with higher body mass index, edematous tissues or subcutaneous emphysema, which are known conditions that limit the visualization by the ultra-sound and consequently make the nerve blockade difficult.^[63,64]^

The knowledge from this study is a statistical confirmation of the previously reported literature that points out the superiority of ACB over FNB in preservation of muscle strength postoperatively, with both the techniques being equally effective in pain control. Physicians can use this piece of knowledge to make evidence based decisions on which peripheral nerve block modality to use with different types of patients undergoing TKA, bearing in mind that pain is a complex multi-dimensional perception that is influenced by several factors above and beyond the pain control method being applied on the patient. These factors include but are not limited to the patient gender, age, length of hospital stay in addition to the familial, psychological, social and cultural variables.^[66,67]^

## 5. Strengths and Limitations

The main strength point of the current systematic review is the high number of included studies in the analysis compared to previous systematic reviews.^[59,68]^ The available data from the included studies allowed for assessment of different outcomes at various time points enriching the analysis.

However, Heterogeneity of the pooled data in different outcomes is a major limitation to this study; this heterogeneity may be explained by the variations in ACB protocols (continuous infusion or single shot) and the different types of anesthesia used in the TKA operation (general or spinal) among the included studies. Variations among patients in pain tolerance may be another source of heterogeneity.^[69]^

## 6. Conclusion

ACB has the advantage of preserving the quadriceps muscle strength and better mobilization after the operation over the FNB, but both the interventions are equal regarding pain control and opioid consumption.

## Author contributions

AA: idea conception, search strategy, screening and extraction conflict resolution and study supervision. EAH, MMM, HAA, EAI, MAH: Screening, data extraction, and writing. AKE, YHA, AAA, ASA: Statistical analysis, manuscript writing and study revision. All authors reviewed the manuscript and approved it for publication.

## Supplementary Material


