# Supplementary material for: Adductor canal block versus femoral nerve block for pain control after total knee arthroplasty: A systematic review and Meta-analysis
Source: Medicine (Baltimore). 2022 Aug 26;101(34):e30110. doi: 10.1097/MD.0000000000030110 (PMC9410636; doi:10.1097/MD.0000000000030110)
Supplement: Supplementary file 1 [file medi-101-e30110-s001.pdf]

Supplementary table 1: Summary of the included studies

Study ID	Study design	Inclusion criteria	Sample size and groups	Intervention	Comparison	Anesthesia (general/spinal)	Results & Adverse Effects	Interventions	
								ACB	FNB
Fahmy et al 2020.(1)	RCT	Patients with osteoarthritis scheduled for TKA.	ACB= 40, FNB= 40	ACB	FNB	Spinal	ACB had a significant higher quadriceps muscle strength and no significant difference regarding ambulation, range of motion and pain estimation.	Ropivacaine 0.5% 20 mL was injected. US guided.	Same procedure but different injection site.
Kac¸maz et al 2021.(2)	RCT	Patients undergoing unilateral knee arthroplasty, Ages of 60 and 75 years.	ACB= 43, FNB= 43	ACB	FNB	Spinal.	No significant results regarding pain control or mobilization.	Injection of 10 ml of 0.5% Bupivacaine, 10 ml of 2% Lidocaine and 2.5 mcg/ml adrenaline via stimulator needle. US guided.	Same procedure but different injection site.
Jaegar et al 2013(3)	RCT	Patients scheduled for primary TKA aged between 50 and 85 years.	ACB=25, FNB=29	ACB	FNB	spinal	Quadriceps strength was significantly higher in the ACB group compared with the FNB group. No difference in pain, or opioid consumption.	Injection of 30 mL of ropivacaine 0.5% via the catheter initially, US guided. Followed by an infusion of 0.2% ropivacaine at a rate of 8 mL/h during the next 24 hours.	same treatment but different injection site
Kim 2013 et al(4)	RCT	elective unilateral TKA, planned combined spinal epidural anesthetic, age 18 to 90 years.	ACB=47, FNB=47	ACB	FNB	spinal	ACB had higher muscle strength at 6 to 8 hours and non-inferior to FNB regarding pain scores and opioid consumption both 24, and 48 hours.	ultrasound-guided ACB (15 cc of 0.5% of bupivacaine with 5 μg/ml epinephrine, via 21-gauge 4-inch Stimuplex A needle)	Ultrasound-guided FNB (30 cc of 0.25% of bupivacaine with 5 μg/ml epinephrine, via 22-gauge 2-inch)
Elkassabny 2016.et al(5)	RCT	Patients scheduled for primary TKA.	ACB=31, FNB=31	ACB	FNB	both	ACB group showed higher muscle strength in manual muscle testing at 24 hours. There was no difference in the risk of falls or ambulation distance.	Ultra-sound guided. A bolus of total volume of 20 mL of ropivacaine 0.5% was injected through the needle.	Ultrasound guided. A bolus of 20 mL ropivacaine 0.5% was injected in increments of 5 mL.
Tan 2018 et al(6)	RCT	Primary unilateral TKA for OA or RA. age more than 18.	ACB=108, FNB=107	ACB	FNB	general	ACB group had higher muscle strength at 24 hours and range of motion at 24, 48 and 72 hours. The total knee pain score was similar between the groups.	ultrasound-guided ACB (20 mL of 0.5% of ropivacaine with 5mg/mL epinephrine, via a 22-gauge 2-inch Stimuplex A needle)	Ultrasound-guided FNB (30 mL of 0.33% of ropivacaine with 5mg/mL epinephrine, via a 22-gauge 2-inch Stimuplex A needle)
Ludwigson 2015 et al(7)	Retrospective study	patients who underwent unilateral TKA August 1, 2012 and March 31, 2014	ACB=148, FNB=149	Single injection ACB	Continuous FNB	both	ACB group showed earlier discharge and better ambulation with no	single injection with ropivacaine or bupivacaine (0.5%, 20 ml)	Injection with ropivacaine or bupivacaine (0.5%, 20 ml). Then continuous infusion of 0.2% bupivacaine

							difference between the two groups in pain management		until the morning of POD one and 0.1% until discontinued on POD 2 at a continuous rate of 5 ml per hour
Seo 2017 et al(8)	Retrospective study	patients over 55 years of age who had undergone primary total knee arthroplasty for osteoarthritis.	ACB=19, FNB=24	continuous ACB	continuous FNB	spinal	ACB showed better muscle strength at days 1,2,3. Better walking ability in days 2,3. No difference between the groups in pain scores or analgesia consumption was observed.	Under ultra-sound guidance 20 mL 0.75% ropivacaine was injected to the adductor canal. Then a 20-gauge catheter was inserted for continuous nerve block.	20 mL 0.75% ropivacaine was injected into the femoral nerve under ultrasound guidance then 20-gauge catheter was inserted for continuous nerve block.
Wiessman 2016 et al(9)	RCT	Patients aged 50–80 years, scheduled for unilateral TKA.	ACB=22, FNB=22	continuous ACB + (ASNB)	continuous FNB+ (ASNB)	general	no difference between the two groups in mobilization ability and pain control.	An anterior sciatic nerve block (ASNB) catheter in combination with CACB then under ultrasound guidance local anesthetic (15 ml of ropivacaine 0.375 %) was injected and the catheter was subsequently inserted.	Uunder ultrasound guidance initial bolus volume of 15 ml of ropivacaine 0.375 % was injected via the catheter. The PCA pump filled with ropivacaine 0.2 % was connected afterwards with a continuous flow rate of 6 ml/h and a bolus function of 6 m. this was preceded by anterior sciatic nerve block.
Klement 2018 et al(10)	Retrospective study	All primary, unilateral total knee arthroplasty patients were identified	ACB=118, FNB=146	ACB + iPACK	FNB + LB-PAI	both	The ACB group had shorter length of stay and more discharges home and less opioid consumption.	A single shot IPACK injection consisted of 20 ml 0.2% ropivacaine with epinephrine. US-guided continuous adductor canal block was performed (An initial bolus dose of 20 ml 0.2% ropivacaine with epinephrine was injected prior to surgery, followed by a 0.2% ropivacaine infusion at eight milliliters per hour postoperatively)	Liposomal bupivacaine periarticular injection with injectable suspension (20 ml vial of 0.25% liposome bupivacaine suspension was diluted to a volume of 40 ml using saline and then injected per protocol) with single shot FNB (under ultrasound with 20 ml of bupivacaine 0.25%)
Mudumbai et al 2013(11)	Retrospective cohort	patients who underwent primary TKA for 1 year.	ACB=66, FNB=102	continuous ACB	continuous FNB	general	the ACB showed higher ambulation distance and the two groups did not differ regarding pain control and opioid consumption	Approximately 20 mL mepivacaine 1.5% was injected into the appropriate compartment to surround the target nerve via the placement needle under ultrasound visualization	Approximately 20 mL mepivacaine 1.5% was injected into the appropriate compartment to surround the target nerve via the placement needle under ultrasound
Machi et al 2015(12)	RCT	adults (≥18 years old) scheduled for primary, unilateral, incompetent knee arthroplasty	ACB=40, FNB=43	ACB	FNB	both	mobilization was better in ACB.	A ropivacaine 0.2% infusion was initiated via perineural catheter with a basal rate of 6 ml/h, a 4-ml bolus, and a lockout of 30 min after insertion of a catheter guided by ultrasound. The basal infusion rate was adjusted in 2 ml/h increments up to twice per day according to pain needs.	A ropivacaine 0.2% infusion was initiated via perineural catheter with a basal rate of 6 ml/h, a 4-ml bolus, and a lockout of 30 min after insertion of a catheter guided by ultrasound. The basal infusion rate was adjusted in 2 ml/h increments up to twice per day according to pain.

Koh et al 2017(13)	RCT	Patients aged <75 years, who were scheduled for same-day bilateral TKA for primary osteoarthritis.	ACB=50, FNB=50	ACB	FNB	general	ACB group showed better muscle strength up to 48 hours then groups were not different. No difference in pain levels is noted.	A local anesthetic, 10 cc of 0.75% ropivacaine (150 mg), was used after insertion of a needle	A local anesthetic, 10 cc of 0.75% ropivacaine (150 mg), was used after insertion of a needle.
Grevstad et al 2014(14)	RCT	unilateral TKA	ACB=25, FNB=25	ACB and saline FNB	FNB and saline ACB	both	ACB group showed better muscle strength compared to baseline after TKA while the muscle strength in FNB declined. ACB improved TUG while pain scores did not differ	150 mL of ropivacaine 0.2% with epinephrine (10µg/mL) under ultrasound guidance in the ACB and saline in FNB	150 mL of ropivacaine 0.2% with epinephrine (10µg/mL). Under ultrasound guidance in FNB and saline in ACB
Shah et al 2014(15)	RCT	primary, unilateral TKA.	ACB=50, FNB=50	continuous ACB	continuous FNB	spinal	ACB group provided better ambulation than FNB and they did not differ regarding pain control and opioid consumption	20 ml of 0.75% ropivacaine was injected to the adductor canal then injection of a further 10 ml of ropivacaine under US guidance. The regimen was 30 cc inj. ropivacaine 0.75% followed by repeated boluses of inj. Ropivacaine 0.25%, 30 cc at an interval of 4 h till 8:00 am on the morning of the second day after surgery (POD2).	30 cc inj. ropivacaine 0.75% followed by repeated boluses of inj. Ropivacaine 0.25%, 30 cc at an interval of 4 h till 8:00 am on the morning of the second day after surgery (POD2). US guided.
Marcinici et al 2016(16)	RCT	40 to 80 years old patients undergoing TKA.	ACB=49, FNB=49	ACB	FNB	general	ACB shows increased muscle strength than FNB and better ambulation by TUG. No difference in pain control or opioid consumption	30 mL of local anesthetic (100 mg of Marcaine) under ultra-sound guidance	30 mL of local anesthetic (100 mg of Marcaine) under ultrasound guidance
lim et al 2019(17)	RCT	patients aged 45–85 years old who were scheduled for primary TKA due to primary osteoarthritis,	ACB=30, FNB=29	ACB	FNB	general	ACB showed better muscle strength after the operation.	For ACB, the block was performed at mid-thigh, midway between the anterior superior iliac spine and patella. The needle approach was from lateral to medial, and local anesthetic was deposited laterally, above, and medially to the artery, between the vaso-adductor membrane and femoral artery.	For FNB, the injection was performed at the level of the inguinal ligament before the bifurcation of the artery. The needle approach was from lateral to medial, and local anesthetic was deposited circumferentially around the nerve
Memtsoudis et al 2014(18)	RCT	Patients scheduled for bilateral TKA	ACB=15, FNB=16	ACB	FNB	spinal	Both interventions did not differ regarding pain control.	ultrasound-guided single-shot ACB	ultrasound-guided single-shot FNB
Zhang wei et al 2014(19)	RCT	patients scheduled for total knee replacement from June 2013 to March	ACB=30, FNB=30	FNB	ACB	spinal	-	A catheter was placed under ultrasound guidance, to locate the adductor canal	A femoral nerve catheter was placed for patients in the femoral nerve block group, the site 1.0 cm below the inguinal

		2014						(approximately 8–12 cm below the inguinal crease).	crease and 1.5 cm distal from the femoral artery was punctured.
Hegazy et al 2014(20)	RCT	Elective unilateral primary TKA age 50–75 years.	ACB=53, FNB=54	ACB	FNB	spinal	-	ultrasound guided ACB	ultrasound guided FNB
patterson et al 2015(21)	Retrospective study	Not mentioned	ACB=39, FNB=41	ACB	FNB	general	-	ACB group received a preoperative ultrasound-guided single-shot ACB,	Not mentioned
mudumbai et al 2015(22)	Retrospective study	Adults (≥ 18 years of age) scheduled for unilateral knee arthroplasty.	ACB=46, FNB=48	ACB	FNB	general	-	perineural catheter insertion under ultrasound guidance	perineural catheter insertion under ultrasound guidance
thacher et al 2017(23)	Retrospective study	Not mentioned	ACB=129, FNB=150	ACB	FNB	Not mentioned	Less episodes of near-fall experience in ACB than FNB	single shot ACB	single shot FNB
Li et al 2016(24)	RCT	patients scheduled for unilateral primary total knee arthroplasty for osteoarthritis with the age of 55 to 80 years old.	ACB=27, FNB=24,	ACB	FNB	Not mentioned	-	Ultrasound-guidance with 20 ml 5 g/L ropivacaine and 0.1 mg adrenaline at the upper thigh.	Ultrasound-guidance with 20 ml 5 g/L ropivacaine and 0.1 mg adrenaline at the upper thigh.
Ardon et al 2015(25)	Retrospective study	Qualifying patients were matched according to age, height, weight, gender.	ACB=45, FNB=45	ACB	FNB	spinal	-	ultrasound-guided technique	ultrasound-guided technique
Rassmussen et al 2014(26)	Retrospective study	patients who underwent unilateral primary TKA by a single surgeon.	ACB=23, FNB=22	ACB	FNB	general	-	Catheter medited	Catheter mediated
thobhani et al 2017(27)	Retrospective study	adult patients (+18 years) who underwent a primary unilateral TKA between September 1, 2014, and January 8, 2015	ACB=22, FNB=25	ACB	FNB	spinal	Better discharge rate and less hospital stay in the ACB group.	US-guided	US-guided

Bolarinwa et al 2018(28)	Retrospective study	patients with diagnosis of primary knee osteoarthritis who underwent primary unilateral TKA.	ACB=791, FNB=834	ACB	FNB	-	-	single shot under ultrasound guidance	single shot under ultrasound guidance
Brennan et al 2018(29)	Retrospective study	patients with osteoarthritis (OA) who received a primary TKA	ACB=141, FNB=104	Continuous ACB	Continuous FNB	-	ACB showed better muscle strength.	Catheter mediated	Catheter mediated
Kukreja et al 2019(30)	RCT	Patients over 18 years of age at the time of surgery.	ACB=45, FNB=45	ACB	FNB	-	Better mobilization in ACB group with no difference in pain control or satisfaction.	Catheter mediated	Catheter mediated
Borys et al 2019(31)	RCT	Inclusion criteria were, age >18 and <75 years, and scheduled for TKA	85	ACB	FNB	spinal	fewer morphine uses in the FNB group than in the ACB group	Catheter mediated	Catheter mediated
Chuan et al 2019(32)	RCT	-.	ACB=75, FNB=76	ACB	FNB	spinal	-	Catheter mediated	Catheter mediated
Wang 2020 et al.(33)	RCT	patients undergoing unilateral, primary TKA were enrolled.	ACB=32, FTB=31	ACB	FTB (femoral triangle block)	general	ACB showed better pain control, analgesia consumption and mobilization.	Ultrasound guided	A liner transducer (6 to 13 MHz, M-Turbo, Sonosite,USA) was used to perform a dynamic scan of the injection site.

ASNP: An anterior sciatic nerve block, ACB: Adductor canal block, FNB: Femoral nerve block, RCT: Randomized controlled trial, iPACK: Interspace between the popliteal artery and capsule of the posterior knee, TKA: Total knee arthroplasty, LB-PAI: liposomal bupivacaine peri-articular injection

1. Fahmy M. EARLY POSTOPERATIVE QUADRICEPS POWER, MOTION RANGE, AND ANALGESIA EVALUATION FOLLOWING KNEE REPLACEMENT AFTER FEMORAL OR ADDUCTOR BLOCK: RANDOMIZED CONTROLLED STUDY. J Musculoskelet Res. 2020 Sep;23(03):2050006.
2. Kaçmaz M, Turhan ZY. The Effect of Femoral Nerve Block and Adductor Canal Block Methods on Patient Satisfaction in Unilateral Knee Arthroplasty: Randomized Non-Inferiority Trial. Geriatr Orthop Surg Rehabil. 2021 Jan;12:215145932199663.
3. Jæger P, Zaric D, Fomsgaard JS, Hilsted KL, Bjerregaard J, Gyrn J, et al. Adductor Canal Block Versus Femoral Nerve Block for Analgesia After Total Knee Arthroplasty. Reg Anesth Pain Med. 2013;38(6):526–32.
4. Kim DH, Lin Y, Goytizolo EA, Kahn RL, Maalouf DB, Manohar A, et al. Adductor Canal Block versus Femoral Nerve Block for Total Knee Arthroplasty: A Prospective, Randomized, Controlled Trial. Anesthesiology. 2014;120(3):540–50.
5. Elkassabany NM, Antosh S, Ahmed M, Nelson C, Israelite C, Badiola I, et al. The Risk of Falls After Total Knee Arthroplasty with the Use of a Femoral Nerve Block Versus an Adductor Canal Block. Anesth Analg. 2016;122(5):1696–703.
6. Tan Z, Kang P, Pei F, Shen B, Zhou Z, Yang J. A comparison of adductor canal block and femoral nerve block after total-knee arthroplasty regarding analgesic effect, effectiveness of early rehabilitation, and lateral knee pain relief in the early stage. Medicine (Baltimore). 2018 Nov;97(48):e13391.
7. Ludwigson JL, Tillmans SD, Galgon RE, Chambers TA, Heiner JP, Schroeder KM. A Comparison of Single Shot Adductor Canal Block Versus Femoral Nerve Catheter for Total Knee Arthroplasty. J Arthroplasty. 2015;30(9):68–71.
8. Seo SS, Kim OG, Seo JH, Kim DH, Kim YG, Park BY. Comparison of the effect of continuous femoral nerve block and adductor canal block after primary total knee arthroplasty. CiOS Clin Orthop Surg. 2017;9(3):303–9.
9. Wiesmann T, Piechowiak K, Duderstadt S, Haupt D, Schmitt J, Eschbach D, et al. Continuous adductor canal block versus continuous femoral nerve block after total knee arthroplasty for mobilisation capability and pain treatment: a randomised and blinded clinical trial. Arch Orthop Trauma Surg. 2016;136(3):397–406.
10. Klement MR, Bullock WM, Nickel BT, Lampley AJ, Seyler TM, Green CL, et al. Continuous adductor canal blockade facilitates increased home discharge and decreased opioid consumption after total knee arthroplasty. Knee. 2019;26(3):679–86.
11. Mudumbai SC, Kim TE, Howard SK, Workman JJ, Giori N, Woolson S, et al. Continuous adductor canal blocks are superior to continuous femoral nerve blocks in promoting early ambulation after TKA. Clin Orthop Relat Res. 2014;472(5):1377–83.
12. Machi AT, Sztain JF, Kormylo NJ, Madison SJ, Abramson WB, Monahan AM, et al. Discharge readiness after tricompartment knee arthroplasty: Adductor canal versus femoral continuous nerve blocks-a dual-center, randomized trial. Anesthesiology. 2015;123(2):444–56.
13. Koh HJ, Koh IJ, Kim MS, Choi KY, Jo HU, In Y. Does Patient Perception Differ Following Adductor Canal Block and Femoral Nerve Block in Total Knee Arthroplasty? A Simultaneous Bilateral Randomized Study. J Arthroplasty. 2017;32(6):1856–61.
14. Grevstad U, Mathiesen O, Valentiner LS, Jaeger P, Hilsted KL, Dahl JB. Effect of Adductor Canal Block Versus Femoral Nerve Block on Quadriceps Strength, Mobilization, and Pain After Total Knee Arthroplasty. Reg Anesth Pain Med. 2015;40(1):3–10.
15. Shah NA, Jain NP. Is continuous adductor canal block better than continuous femoral nerve block after total knee arthroplasty? Effect on ambulation ability, early functional recovery and pain control: A randomized controlled trial. J Arthroplasty. 2014;29(11):2224–9.
16. Macrinici GI, Murphy C, Christman L, Drescher M, Hughes B, Macrinici V, et al. Prospective, Double-Blind, Randomized Study to Evaluate Single-Injection Adductor Canal Nerve Block Versus Femoral Nerve Block. Reg Anesth Pain Med. 2017;42(1):10–6.
17. Lim YC, Quek HYK, Phoo WHJ, Mah CL, Tan S. A randomised controlled trial comparing adductor canal block and femoral nerve block for knee arthroplasty. Singapore Med J. 2019;60(3):145–9.
18. Memtsoudis SG, Yoo D, Stundner O, Danninger T, Ma Y, Poultsides L, et al. Subsartorial adductor canal vs femoral nerve block for analgesia after total knee replacement. Int Orthop. 2015;39(4):673–80.
19. Zhang W, Hu Y, Tao Y, Liu XB, Wang G. Ultrasound-guided continuous adductor canal block for analgesia after total knee replacement. Chin Med J (Engl). 2014;127(23):4077–81.
20. Hegazy N, Sultan S. Comparison between effects of adductor canal block and femoral nerve block on early postoperative course in total knee arthroplasty: A prospective double-blind, randomized controlled study. Ain-Shams J Anaesthesiol. 2015;8(1):124.

21. Patterson ME, Bland KS, Thomas LC, Elliott CE, Soberon JR, Nossaman BD, et al. The adductor canal block provides effective analgesia similar to a femoral nerve block in patients undergoing total knee arthroplasty - A retrospective study. J Clin Anesth. 2015;27(1):39–44.

22. Mudumbai SC, Ganaway T, Kim TE, Howard SK, Giori NJ, Shum C, et al. Can bedside patient-reported numbness predict postoperative ambulation ability for total knee arthroplasty patients with nerve block catheters? Korean J Anesthesiol. 2016;69(1):32–6.

23. Thacher RR, Hickernell TR, Grosso MJ, Shah R, Cooper HJ, Maniker R, et al. Decreased risk of knee buckling with adductor canal block versus femoral nerve block in total knee arthroplasty: a retrospective cohort study. Arthroplast Today. 2017;3(4):281–5.

24. Li D, Tan Z, Kang P, Shen B, Pei F. Effects of multi-site infiltration analgesia on pain management and early rehabilitation compared with femoral nerve or adductor canal block for patients undergoing total knee arthroplasty: a prospective randomized controlled trial. Int Orthop. 2017;41(1):75–83.

25. Ardon AE, Clendenen SR, Porter SB, Robards CB, Greengrass RA. Opioid consumption in total knee arthroplasty patients: A retrospective comparison of adductor canal and femoral nerve continuous infusions in the presence of a sciatic nerve catheter. J Clin Anesth. 2016;31:19–26.

26. Rasmussen M, Kim E, Kim TE, Howard SK, Mudumbai S, Giori NJ, et al. A retrospective comparative provider workload analysis for femoral nerve and adductor canal catheters following knee arthroplasty. J Anesth. 2015;29(2):303–7.

27. Thobhani S, Scalercio L, Elliott CE, Nossaman BD, Thomas LC, Yuratich D, et al. Novel regional techniques for total knee arthroplasty promote reduced hospital length of stay: An analysis of 106 patients. Ochsner J. 2017;17(3):233–8.

28. Bolarinwa SA, Novicoff W, Cui Q. Reducing costly falls after total knee arthroplasty. World J Orthop. 2018 Oct;9(10):198–202.

29. Brennan PT, Villa JM, Rossi MD, Sanchez-Gonzalez MA, Lavernia CJ. Rehabilitation Outcomes for Total Knee Arthroplasties: Continuous Adductor Canal Block Versus Continuous Femoral Nerve Block. Geriatr Orthop Surg Rehabil. 2018;9:215145851875619.

30. Kukreja P, Bevinetto C, Brooks B, McKissack H, Montgomery TP, Alexander B, et al. Comparison of Adductor Canal Block and Femoral Nerve Block for Early Ambulation After Primary Total Knee Arthroplasty: A Randomized Controlled Trial. Cureus. 2019;11(12).

31. Borys M, Domagała M, Wencław K, Jarczyńska-Domagała J, Czuczwar M. Continuous femoral nerve block is more effective than continuous adductor canal block for treating pain after total knee arthroplasty. Medicine (Baltimore). 2019;98(39):e17358.

32. Chuan A, Lansdown A, Brick KL, Bourgeois AJG, Pencheva LB, Hue B, et al. Adductor canal versus femoral triangle anatomical locations for continuous catheter analgesia after total knee arthroplasty: a multicentre randomised controlled study. Br J Anaesth. 2019;123(3):360–7.

33. Wang C-G, Ding Y-L, Wang Y-Y, Liu J-Y, Zhang Q. Comparison of Adductor Canal Block and Femoral Triangle Block for Total Knee Arthroplasty. Clin J Pain. 2020 Jul;36(7):558–61.
