# Supplementary material for: Adductor canal block versus femoral nerve block for pain control after total knee arthroplasty: A systematic review and Meta-analysis
Source: Medicine (Baltimore). 2022 Aug 26;101(34):e30110. doi: 10.1097/MD.0000000000030110 (PMC9410636; doi:10.1097/MD.0000000000030110)
Supplement: Supplementary file 2 [file medi-101-e30110-s002.pdf]

Supplementary table 2: Risk of bias assessment of cohort (prospective and retrospective) studies according to the national institute of health (NIH) quality assessment tool (Yes, No, not applicable (NA), cannot determine (CD), or not reported (NR))

Studies	1. Was the research question or objective in this paper clearly stated?	2. Was the study population clearly specified and defined?	3. Was the participation rate of eligible persons at least 50%?	4. Were all the subjects selected or recruited from the same or similar populations? Were inclusion and exclusion criteria for being in the study pre-specified and applied uniformly to all participants?	5. Was a sample size justification, power description, or variance and effect estimates provided?	6. For the analyses in this paper, were the exposure(s) of interest measured prior to the outcome(s) being measured?	7. Was the time frame sufficient so that one could reasonably expect to see an association between exposure and outcome if it existed?	8. For exposures that can vary in amount or level, did the study examine different levels of the exposure as related to the outcome (e.g., categories of exposure, or exposure measured as continuous variable)?	9. Were the exposure measures (independent variables) clearly defined, valid, reliable, and implemented consistently across all study participants?	10. Was the exposure(s) assessed more than once over time?	11. Were the outcome measures (dependent variables) clearly defined, valid, reliable, and implemented consistently across all study participants?	12. Were the outcome assessors blinded to the exposure status of participants?	13. Was loss to follow-up after baseline 20% or less?	14. Were key potential confounding variables measured and adjusted statistically for their impact on the relationship between exposure(s) an outcome(s)?	Total scores: Yes = 1, No = 0.5, NR & NA & CD = 0	Quality rating: good (14-12 point) or fair (7-11 point) or poor (6-0 points)
**Ludwigson et al**	Yes	No	NA	Yes	No	Yes	Yes	No	Yes	NA	Yes	No	Yes	No	9.5	fair quality
**seo et al**	Yes	Yes	NA	Yes	No	Yes	Yes	No	Yes	NA	Yes	No	Yes	No	10	fair quality
**klement et al**	Yes	Yes	NR	Yes	No	Yes	No	No	Yes	NA	Yes	No	Yes	Yes	9.5	fair quality
**Mudumbai et al 2013**	Yes	Yes	Yes	Yes	Yes	No	Yes	No	Yes	NA	Yes	No	Yes	Yes	11.5	Good quality
**patterson et al**	Yes	Yes	Yes	Yes	NR	No	No	No	Yes	NA	Yes	No	Yes	No	9.5	Fair quality
**mudumbai et al 2016**	Yes	Yes	NR	Yes	NR	No	No	No	Yes	NA	Yes	No	Yes	Yes	8.5	Fair quality
**Thacher et al**	Yes	Yes	No	Yes	NR	NR	Yes	No	Yes	NA	Yes	No	Yes	No	9	Fair quality
**Ardon et al**	Yes	Yes	Yes	Yes	Yes	Yes	Yes	NA	Yes	NA	Yes	No	Yes	No	11	fair quality
**Bolarinwa et al**	Yes	Yes	Yes	Yes	NR	NR	Yes	NA	Yes	NA	Yes	No	Yes	No	9	Fair quality

**Brennan et al**	Yes	Yes	NR	Yes	No	Yes	Yes	NA	Yes	NA	Yes	No	Yes	No	9.5	Fair quality
**Rassmussen et al**	Yes	Yes	No	Yes	No	Yes	NR	NA	Yes	NA	Yes	No	Yes	No	8	Fair quality
**thobhani et al**	Yes	Yes	Yes	Yes	NR	NR	Yes	Yes	Yes	NA	Yes	No	Yes	No	10	Fair quality

NA: not applicable, NR: not reported, CD: cannot determine
