# Supplementary figures and images for: Adductor canal block versus femoral nerve block for pain control after total knee arthroplasty: A systematic review and Meta-analysis
Source: Medicine (Baltimore). 2022 Aug 26;101(34):e30110. doi: 10.1097/MD.0000000000030110 (PMC9410636; doi:10.1097/MD.0000000000030110)

Supplementary figure 1: Pain Score at 6-8 hours

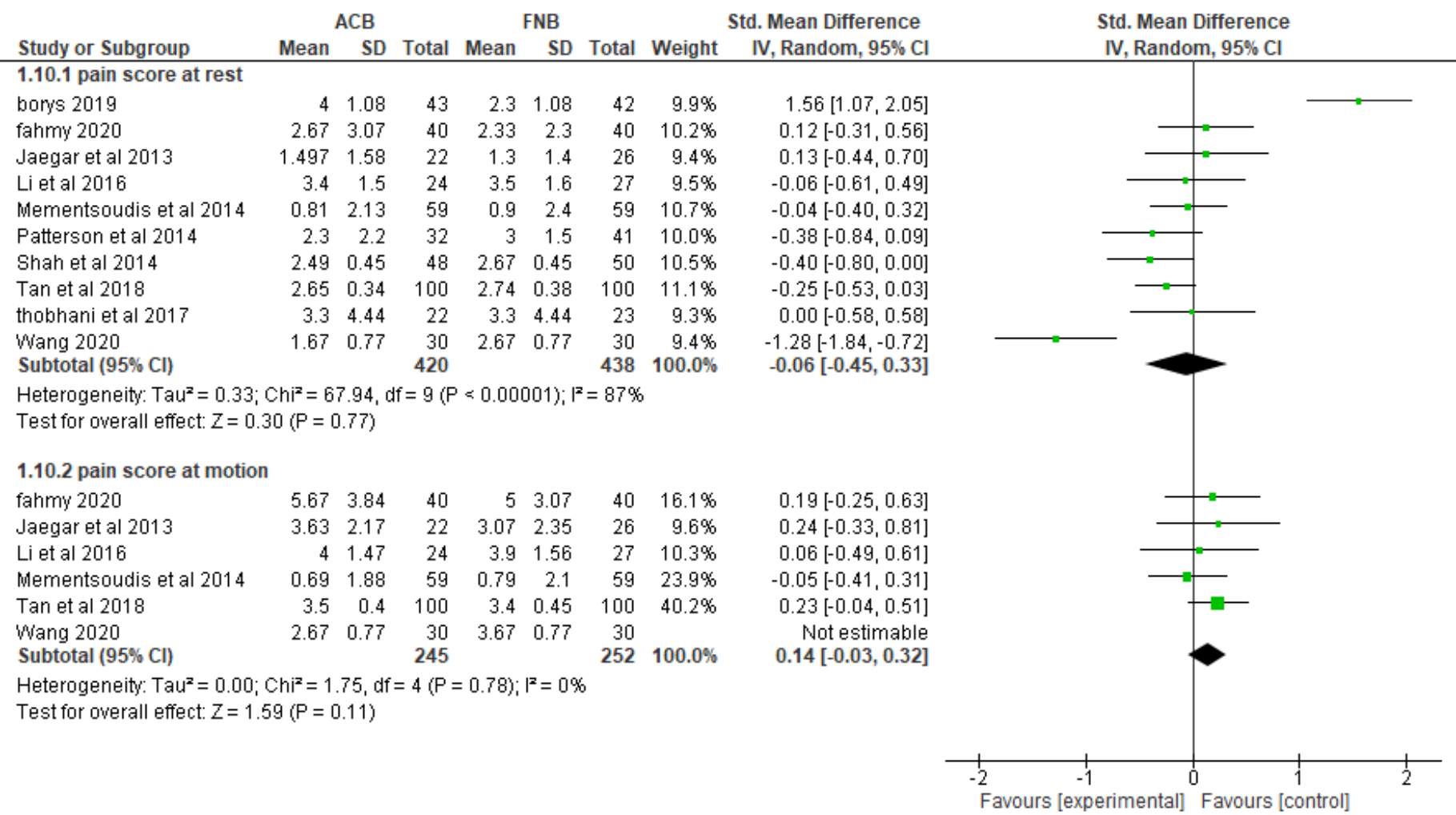

Supplement: Supplementary file 3 [file medi-101-e30110-s003.pdf]

Supplementary figure 2: Pain Score at 48 hours

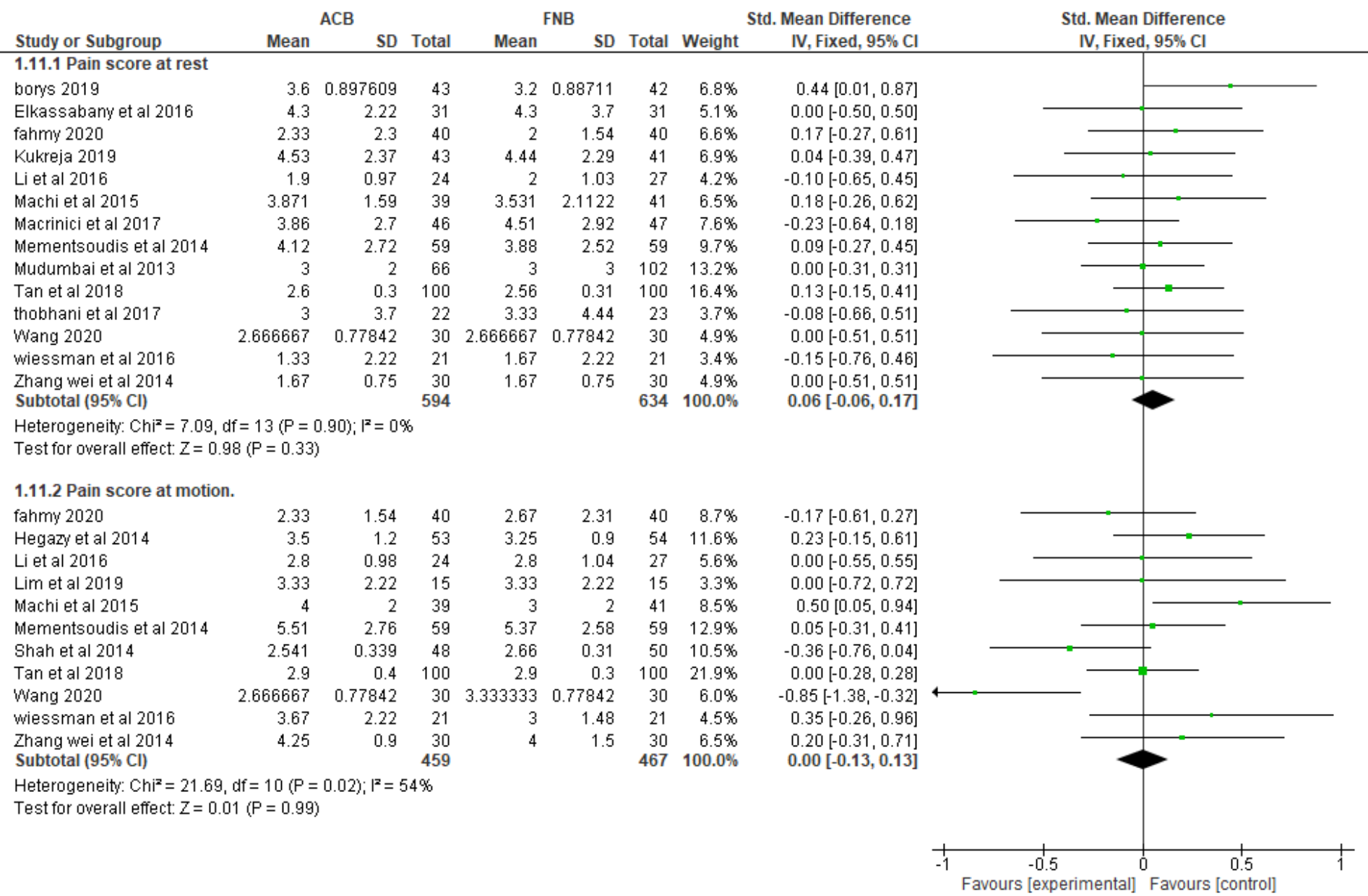

Supplement: Supplementary file 4 [file medi-101-e30110-s004.pdf]

Supplementary figure 3: Quadriceps muscle strength 2 days postoperatively.

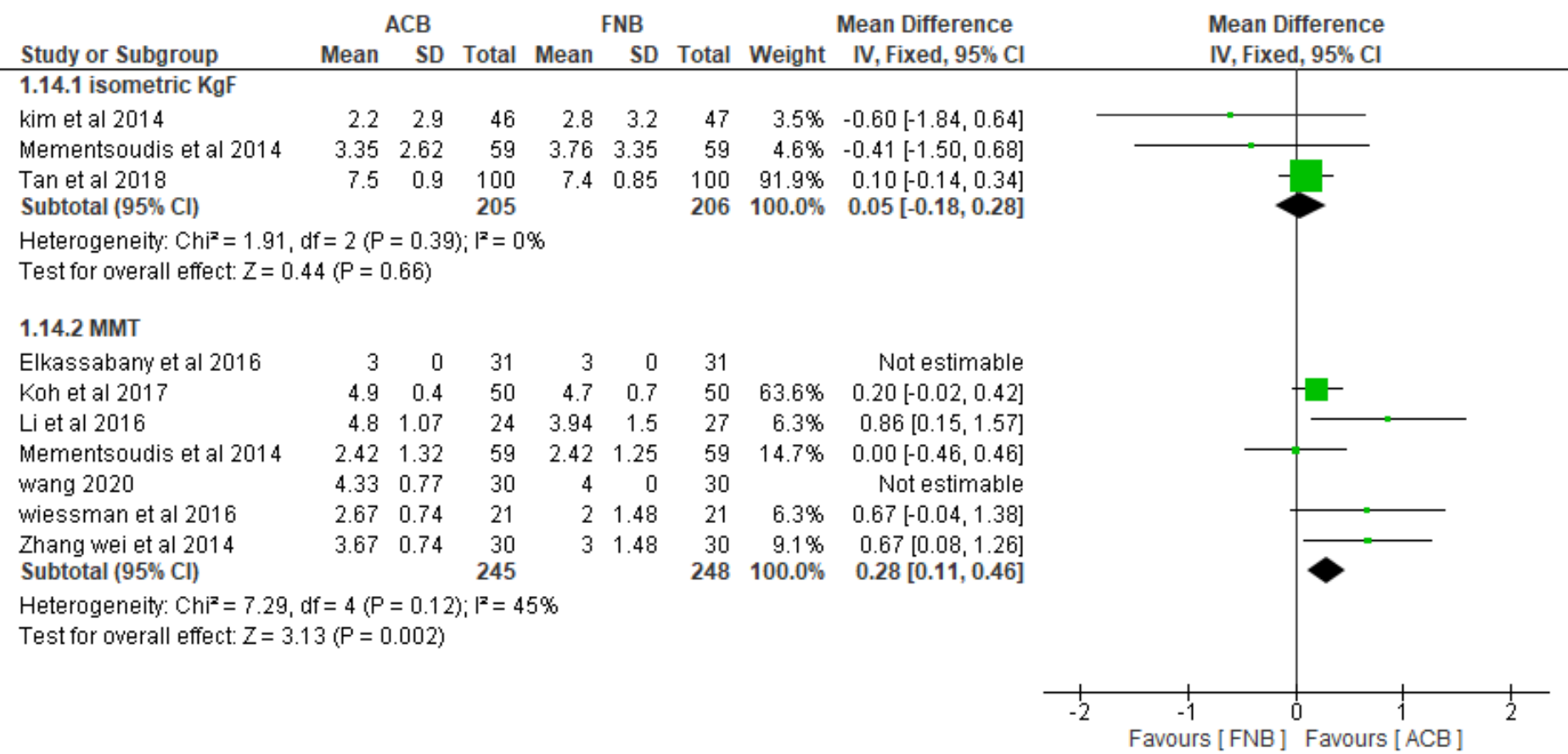

Supplement: Supplementary file 5 [file medi-101-e30110-s005.pdf]

Supplementary figure 4: Mobilization by ambulation and walking distance.

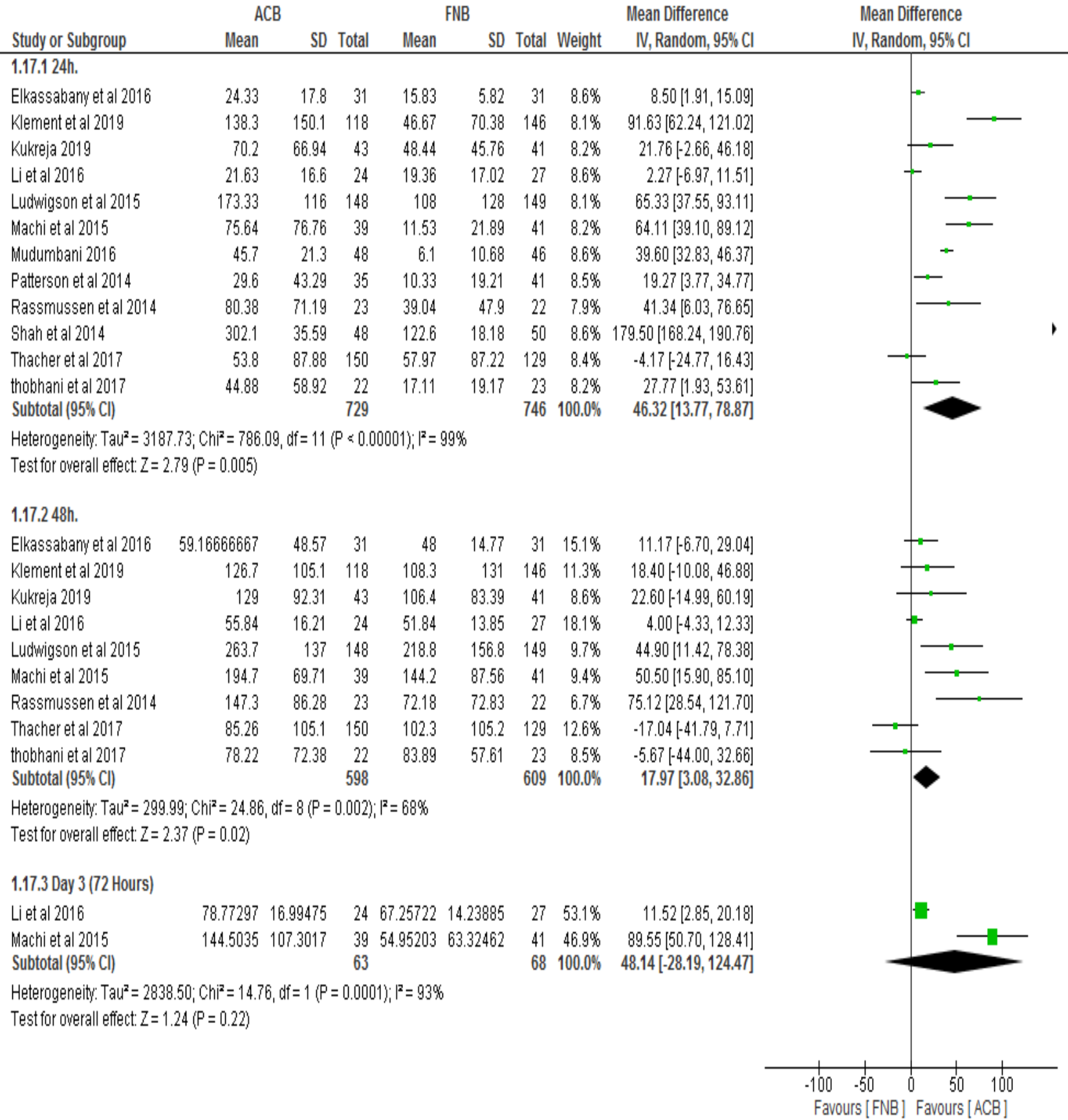

Supplement: Supplementary file 6 [file medi-101-e30110-s006.pdf]

Supplementary figure 5: Length of hospital stay.

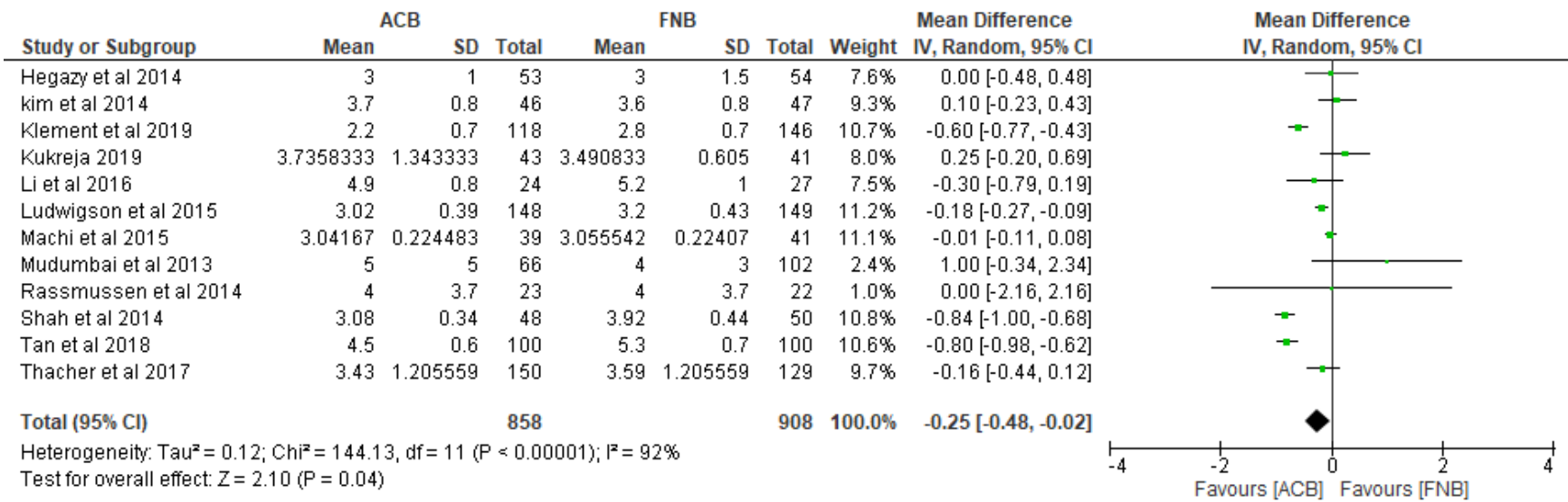

Supplement: Supplementary file 7 [file medi-101-e30110-s007.pdf]

Supplementary figure 6: Risk of falls.

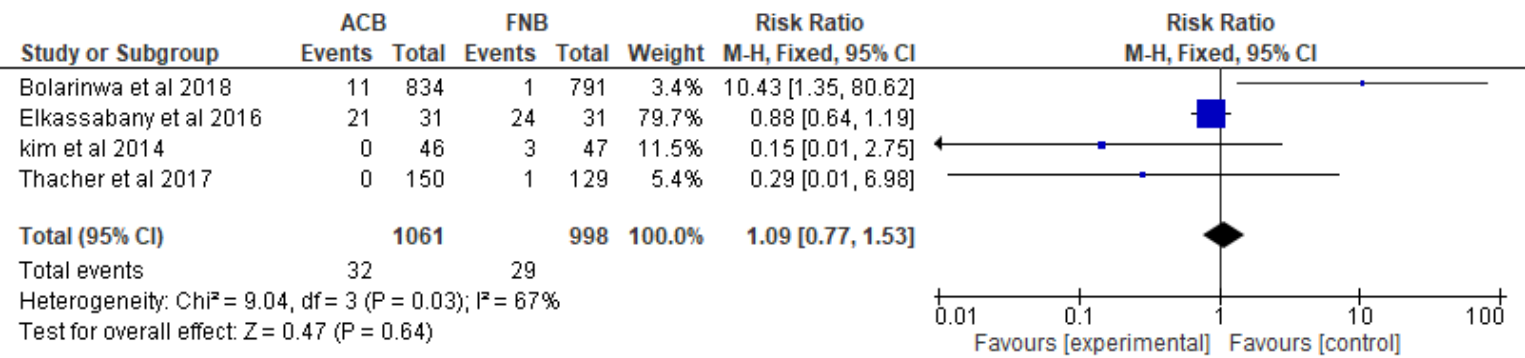

Supplement: Supplementary file 8 [file medi-101-e30110-s008.pdf]

Supplementary figure 7: Mean patient satisfaction.

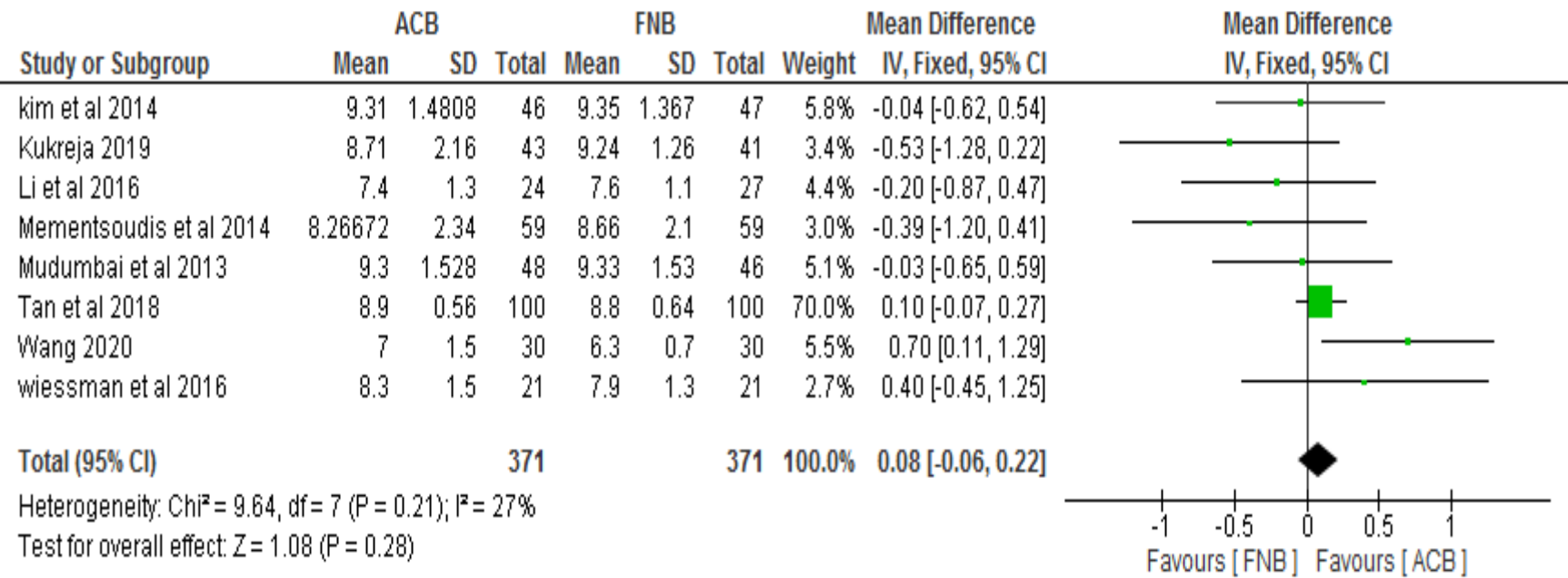

Supplement: Supplementary file 9 [file medi-101-e30110-s009.pdf]
